# Enhancing Diagnostic Yield: The Role of Endobronchial Ultrasound-Guided Cryobiopsy in the Evaluation of Mediastinal Lymphadenopathy

**DOI:** 10.1016/j.chpulm.2026.100254

**Published:** 2026-03-09

**Authors:** Nihar Shah, Roshen Mathew, Hasnain Bawaadam, Arthur Oliver Romero, Sushant Nanavati, Stephanie Baltaji, Toribiong Uchel, Wes Shepherd, Jon Brandon Mullholand, Josh Boron, Elizabel Thomas, Cliff Chen, Danai Khemasuwan

**Affiliations:** a Department of Interventional Pulmonology, Inova Schar Cancer Institute, Inova Fairfax Hospital, Falls Church, VA; b Interventional Pulmonology Services, WVU Medicine Camden Clark Medical Center, Parkersburg, WV; c Department of Interventional Pulmonology, Aurora Medical Center Kenosha, Kenosha, WI; d Division of Pulmonary and Critical Care, Kerkorian School of Medicine at UNLV, Las Vegas, NV; e Department of Interventional Pulmonology, Allegheny Health Network, Pittsburgh, PA; f Departments of Interventional Pulmonology, Virginia Commonwealth University, Richmond, VA; g Pulmonology and Critical Care Medicine, Virginia Commonwealth University, Richmond, VA; h Internal Medicine, Virginia Commonwealth University, Richmond, VA; i Department of Internal Medicine, WVU Medicine Camden Clarke Medical Center, Parkersburg, WV; j Department of Pulmonology and Critical Care Medicine, University of Nevada, Las Vegas, NV

**Keywords:** cryobiopsy, EBUS-MCB, EBUS-TBNA

## Abstract

**Background:**

Endobronchial ultrasound-guided transbronchial needle aspiration (EBUS-TBNA) is widely used for evaluating mediastinal lymphadenopathy, particularly in lung cancer diagnosis. However, its diagnostic effectiveness may be limited for conditions such as granulomatous disease and lymphoma, which often require preserved tissue architecture. Endobronchial ultrasound-guided mediastinal cryobiopsy (EBUS-MCB) is an emerging technique with promising diagnostic potential. This study evaluates the additive diagnostic yield of EBUS-MCB in patients with inconclusive or inadequate rapid on-site evaluation (ROSE) during EBUS-TBNA.

**Research Question:**

Does the addition of EBUS-MCB improve diagnostic yield in patients with inconclusive or absent ROSE during EBUS-TBNA, and is the technique safe in clinical practice?

**Study Design and Methods:**

We conducted a retrospective multicenter study of patients with inconclusive or inadequate ROSE during EBUS-TBNA. EBUS-MCB was performed in addition to standard transbronchial needle aspiration sampling. Diagnostic yield and procedural complications were assessed.

**Results:**

Among 145 patients, EBUS-MCB was performed after inconclusive ROSE. Most procedures were conducted under general anesthesia (98.6%), with endotracheal intubation in 71.7%. The mean lymph node diameter was 20.95 mm, with an average of 5.1 transbronchial needle aspiration passes and 3.6 cryobiopsy samples per node. EBUS-TBNA alone achieved a diagnostic yield of 91.7%, identifying 34 malignancies. EBUS-MCB detected 15 additional malignancies, increasing the total to 49 and raising overall diagnostic yield to 97.7%. EBUS-MCB also identified 25 additional granulomatous cases beyond the 29 detected by EBUS-TBNA. A definitive diagnosis of malignancy or granulomatous disease was established in 106 patients (73.1%). No major complications occurred; minor bleeding was managed conservatively.

**Interpretation:**

Our results show that EBUS-MCB identified additional diagnoses of granulomatous disease and lymphoma in patients with inconclusive ROSE after EBUS-TBNA and appears to be a safe and effective adjunctive technique for enhancing diagnostic yield in mediastinal lymphadenopathy.

**Take-Home Points**

**Study Question:** Can endobronchial ultrasound-guided mediastinal cryobiopsy (EBUS-MCB) improve diagnostic yield in patients with mediastinal lymphadenopathy when endobronchial ultrasound-guided transbronchial needle aspiration (EBUS-TBNA) and rapid on-site evaluation are inconclusive?

**Results:** In a multicenter US cohort of 145 patients, EBUS-MCB increased overall diagnostic yield from 91.7% with EBUS-TBNA alone to 97.9%, particularly improving detection of lymphoma and granulomatous disease, without major complications.

**Interpretation:** Our results suggest that EBUS-MCB is a safe and effective adjunct to EBUS-TBNA, especially valuable for challenging cases such as lymphoma and granulomatous disorders, and should be considered in centers where rapid on-site evaluation or transbronchial needle aspiration alone prove inadequate.

Endobronchial ultrasound-guided transbronchial needle aspiration (EBUS-TBNA) has revolutionized the evaluation of mediastinal lymphadenopathy, becoming a cornerstone for diagnosing lung cancer and other thoracic malignancies. This minimally invasive approach is favored for the better safety profile and diagnostic yield equivalent to mediastinoscopy.^1^ With the increasing approval of targeted therapies for the treatment of lung cancer, it is incumbent on the bronchoscopist to obtain sufficient lymph node tissue not only for the diagnosis of malignancy but also to ensure adequacy for comprehensive molecular testing. Among these molecular tests are those required for next-generation sequencing (NGS). The NGS panels using EBUS-TBNA samples in nonsquamous non-small cell cancer demonstrated high success rates (92.5% for DNA and 91.5% for RNA). Higher tumor cellularity and advanced nodal stages (N2/N3) were associated with better DNA NGS performance.^2^^,^^3^

A limitation of EBUS-TBNA is making a diagnosis in conditions where larger tissue samples allow demonstration of histologic structure, such as lymphoma and granulomatous disease.^4^, ^5^, ^6^ Lymphoid neoplasms with smaller characteristic cells and distinct immunophenotypic profiles, such as mantle cell lymphoma and T-cell lymphoblastic lymphoma, showed high diagnostic sensitivity and reproducibility with small sample size. In contrast, other subtypes like follicular lymphoma, marginal zone lymphoma, and Hodgkin lymphoma, which require architectural evaluation or identification of cellular subpopulations, were challenging to diagnose with small-volume samples.^7^ The endobronchial ultrasound-guided mediastinal cryobiopsy (EBUS-MCB) has a potential role in diagnosis of lymphoma with larger samples. The diagnostic yield of lymphoma of EBUS-MCB ranges from 87% to 100%.^8^

The adoption of EBUS-MCB has been growing, particularly in Asia and Europe, where most of the reported clinical experiences have originated. This technique offers a promising minimally invasive alternative for acquiring larger tissue samples from mediastinal lymph nodes, addressing the limitations of EBUS-TBNA. However, in North America, the adoption of EBUS-MCB remains primarily confined to academic institutions. The barriers to its widespread implementation include a lack of standardized technique, concerns about procedural costs, and limited published data pertaining additive diagnostic yield and safety, which largely consist of isolated case reports. These factors have hindered the integration of EBUS-MCB into routine community-based pulmonary practice.

To address this gap, we conducted a retrospective study of EBUS-MCB cases performed at 4 centers in the US, including both academic and community-based medical institutions. These cases involved scenarios where rapid on-site evaluation (ROSE) results were inconclusive, inadequate for diagnostic purposes, or unavailable, highlighting the utility of EBUS-MCB in challenging clinical situations.^9^ By capturing variations in geographic locations and practice environments, we aim to establish the feasibility, safety, and generalizability of EBUS-MCB in real-world clinical practice, emphasizing its potential for broader adoption in community care settings.

## Study Design and Methods

### Patient Selection and Study Design

This study included all patients undergoing EBUS-TBNA for undiagnosed mediastinal lymphadenopathy identified on CT scan or PET-CT scan from October 2022 to September 2024. Institutional review board approval was obtained from Virginia Commonwealth Univeristy (2108 & HM20025491) before initiating the study. The research was conducted across 4 diverse institutions in the US: Virginia Commonwealth University (Virginia), West Virginia University Camden Clark Medical Center (West Virginia), University Medical Center of Southern Nevada (Nevada), and Advocate Aurora Medical Center Kenosha (Wisconsin), ensuring representation of both academic and community-based practice settings.

Patients underwent EBUS-TBNA using the Olympus BF-UC180F bronchoscope under general anesthesia with either endotracheal intubation or laryngeal mask airway, depending on institutional protocol. Informed consent was obtained from all participants in accordance with standard clinical practice. All procedures were performed by board-certified, fellowship-trained interventional pulmonologists. At the academic medical center (Virginia Commonwealth University), which has an interventional pulmonology fellowship program, EBUS-MCB procedures were conducted jointly by interventional pulmonology board-certified attendings and fellows. Note that each of the participating operators had > 150 endobronchial ultrasound (EBUS) procedures performed yearly at their sites. During each procedure, endosonographic assessment of the mediastinal lymph nodes was performed, also with color Doppler for any blood vessels transvering the lymph node if hypoechoic areas were noted. This was followed by EBUS-TBNA using a 21/22-gauge needle (Olympus). The number of mediastinal lymph node biopsies per patient was recorded for analysis. All the participating sites had ROSE available for all cases. ROSE of the aspirated material was performed by pulmonary cytopathologists based on institutional practices. Aspirated material was prepared as slides for ROSE evaluation, whereas the remainder was collected in Roswell Park Memorial Institute medium for cell block preparation. Flow cytometry was performed selectively in cases with clinical, radiographic, or cytologic suspicion of lymphoproliferative disease, at the discretion of the cytopathologist, or in patients with a prior history of lymphoma. In cases where ROSE results were inconclusive or deemed inadequate as per adequacy assessment from ROSE, cryobiopsy of the largest lymph node was performed. This approach ensured comprehensive evaluation and allowed for an innovative integration of EBUS-MCB in routine diagnostic workflows.

### EBUS-MCB Procedure

**Figure 1:**
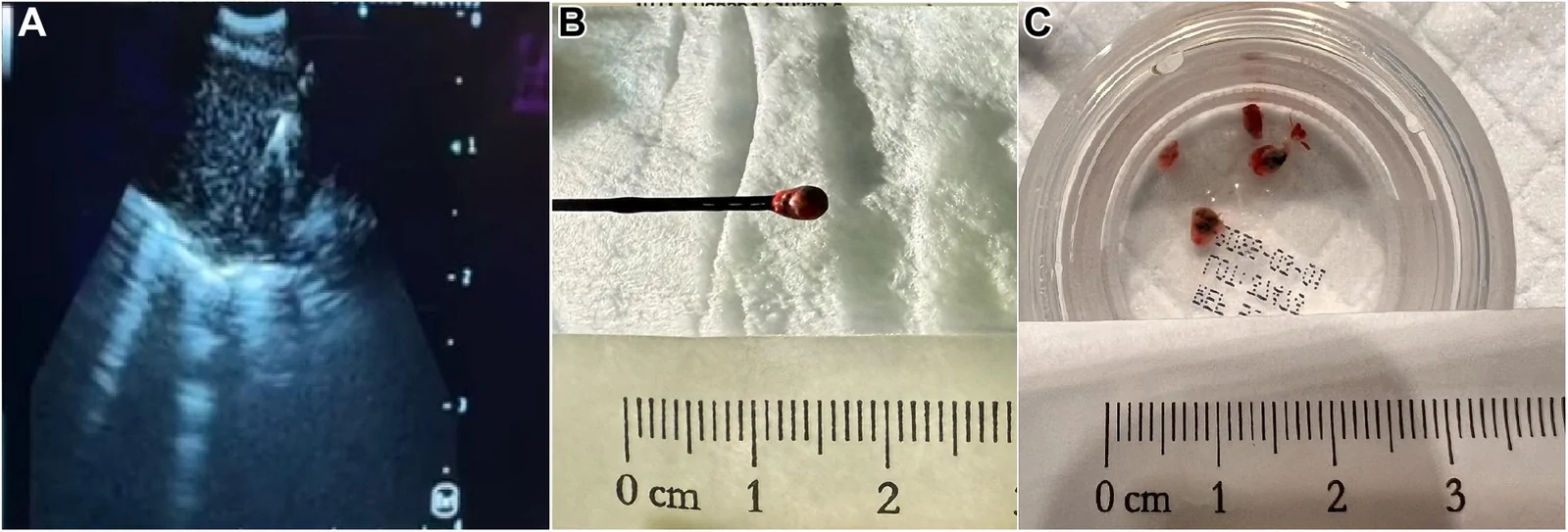
A, Direct visualization of the probe’s tip embedded within the lymph node, (B) a freeze tissue biopsy on 1.1-mm cryoprobe, and (C) tissue pieces in formalin.

The procedure for performing EBUS-MCB involved a carefully orchestrated series of steps to ensure precision and safety. All EBUS-MCB procedures were done on lymph nodes ≥ 15 mm in size. Initially, an EBUS-TBNA needle was used to puncture the bronchial wall at the target site, creating a weakened area to facilitate subsequent cryoprobe passage. After this, a 1.1-mm cryoprobe (ERBE) was inserted through the working channel of the EBUS bronchoscope. The cryoprobe was then advanced gently through the previously punctured tract into the mediastinal lymph node, with its position confirmed in real time by direct visualization of the probe’s tip embedded within the lymph node (Fig 1).

Once properly positioned, the cryoprobe was activated for 5 to 6 seconds to freeze the target tissue. The frozen specimen, securely adhered to the cryoprobe, was retrieved en bloc along with the bronchoscope. The tissue samples were immediately thawed in saline before being fixed in formalin to preserve cellular and architectural integrity. A minimum of 3 cryobiopsy specimens were collected per case to maximize diagnostic yield. All procedures were performed in a dedicated ambulatory endoscopy unit, and complications, if any, were meticulously recorded. A sample was deemed diagnostic if final pathology provided a definitive diagnosis, eliminating the need for additional diagnostic procedures. Nondiagnosis was defined as insufficient material to reach a conclusive diagnosis. This meticulous protocol ensured both procedural safety and optimal diagnostic outcomes. The definition of diagnostic yield was including benign diagnoses, which was referred to as “liberal” by Leonard et al.^10^

### Statistical Analysis

Continuous variables, such as patient age, lymph node size, and tumor cellularity percentage, were summarized using mean values alongside their SDs and medians, along with their corresponding interquartile ranges. This approach ensures that variability within the data is clearly represented, offering insights into the spread and consistency of the measured parameters. Categorical variables, such as patient sex, nodal stage, presence of complications, number of biopsies, and the adequacy of cryobiopsy samples for diagnosis, were expressed as percentages. This method provides an intuitive view of the distribution of these variables, enabling readers to grasp the proportion of patients or procedures falling into each category. McNemar test was used to compare the diagnostic yield between transbronchial needle aspiration (TBNA) alone and combined TBNA + mediastinal cryobiopsy (MCB), accounting for the paired nature of the data where each patient underwent both sampling methods sequentially. Statistical significance was set at a 2-sided alpha level of .05.

Given the retrospective design, no prior sample size or power calculation was performed. To provide context for interpretation, we report a post hoc detectable effect size for paired proportions based on the observed sample size.

## Results

In this cohort, 145 patients underwent EBUS-TBNA of mediastinal and hilar lymph nodes, with inconclusive or inadequate ROSE during the procedure, leading to subsequent EBUS-MCB in all patients. Most EBUS-MCB procedures (98.6%) were performed under general anesthesia. Regarding airway management, 71.7% of patients were intubated with an endotracheal tube, whereas 28.3% were managed with a laryngeal mask airway. PET scans were available for 54.5% of the participants. Among those with PET imaging, 83.5% demonstrated increased FDG uptake in at least one of the target lymph nodes, indicating metabolic activity suggestive of malignancy or other pathologic processes.

Across 145 patients, a total of 318 lymph nodes were biopsied using EBUS-TBNA, with an average of at least 2 lymph nodes sampled per patient, before attempting EBUS-MCB in 145 lymph nodes (1 per patient). EBUS-MCB was performed in all patients, only because ROSE was inadequate or inconclusive. The demographic and lymph node characteristics of the study population are summarized in Table 1. The mean diameter of the lymph nodes sampled by EBUS-MCB, measured along the longest axis, was 20.95 ± 7.4 mm. On average, 5.12 ± 1.9 passes were performed using EBUS-TBNA at the same lymph node target where EBUS-MCB was subsequently attempted. The most commonly sampled lymph node by EBUS-MCB was station 7 (53.7%). The details pertaining to the frequency of EBUS-MCB at various lymph node stations are included in Table 2. The mean number of cryobiopsy samples obtained per lymph node was 3.62 ± 1.0. The average tissue size obtained through EBUS-MCB was 9 ± 1.5 mm.

**Table 1:** Demographic Data (N = 145)

Parameter	Value
Age, y	59.35 [14.5]
Sex	
Female	49.6 (72/145)
PET scan availability	54.5 (79/145)
PET-avid LN (for PET scan available cases)	83.5 (66/79)
Sedation (GA)	98.6 (143/145)
Airway type (ET tube)	71.7 (104/145)

Data are presented as mean [SD] or % (No./total No.). ET = endotracheal; GA = general anesthesia; LN = lymph node.

**Table 2:** Number of Mediastinal Lymph Node Biopsies by EBUS-TBNA Only and Sequential EBUS-MCB (N = 145)

Lymph Node Stations	EBUS-TBNA Only	Sequential EBUS-MCB
No. of lymph nodes	318	145
2R	2 (0.6)	2 (1.4)
4R	72 (22.6)	20 (14)
4L	16 (5)	3 (2)
7	122 (38.4)	78 (53.7)
11L	43 (13.5)	11 (7.5)
11R	55 (17.3)	13 (8.9)
12L	1 (0.3)	1 (0.7)
12R	1 (0.3)	0 (0)
Tumor mass	6 (1.8)	8 (5.5)
Two lymph node targets	Not applicable	9 (6.2)

Data are presented as No. (%). EBUS-MCB = endobronchial ultrasound-guided mediastinal cryobiopsy; EBUS-TBNA = endobronchial ultrasound-guided transbronchial needle aspiration.

**Figure 2:**
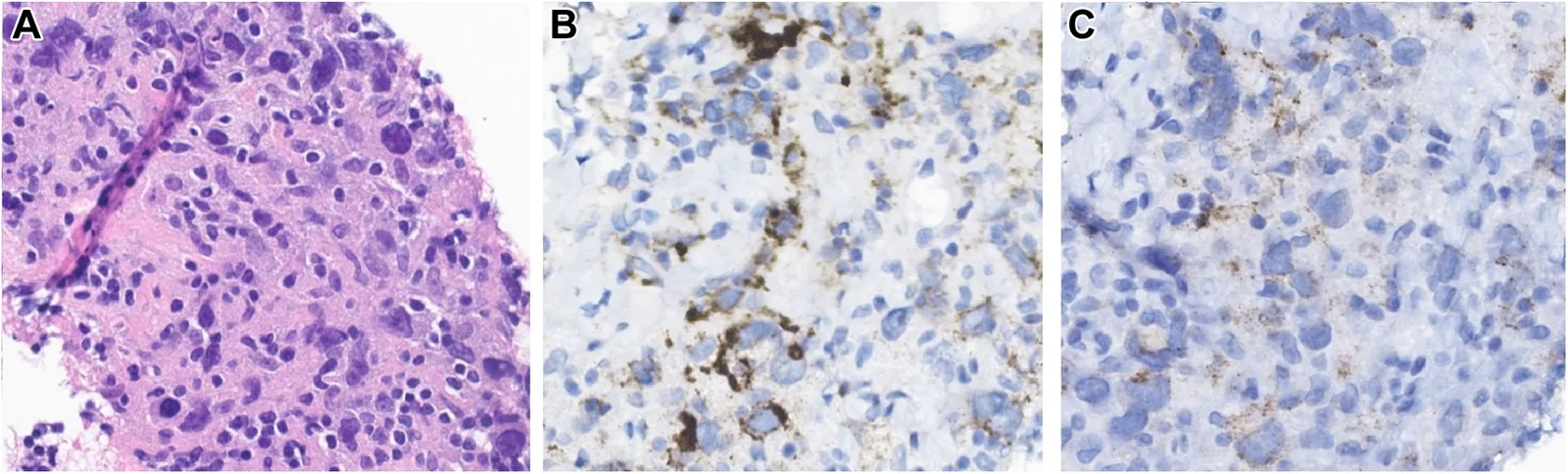
A, A lymph node biopsy with a disrupted architecture, showing a mixed inflammatory infiltrate of small lymphocytes, histiocytes, eosinophils, and scattered large, atypical cells. (A) Hematoxylin & eosin stain, magnification panel A: 200x. The immunohistochemistry stains used to diagnose Hodgkin lymphoma are CD30 (B) and CD15 (C), magnification panel B,C : 300x. (Image courtesy of pathologist, Guanhua Lai, MD.)

**Figure 3:**
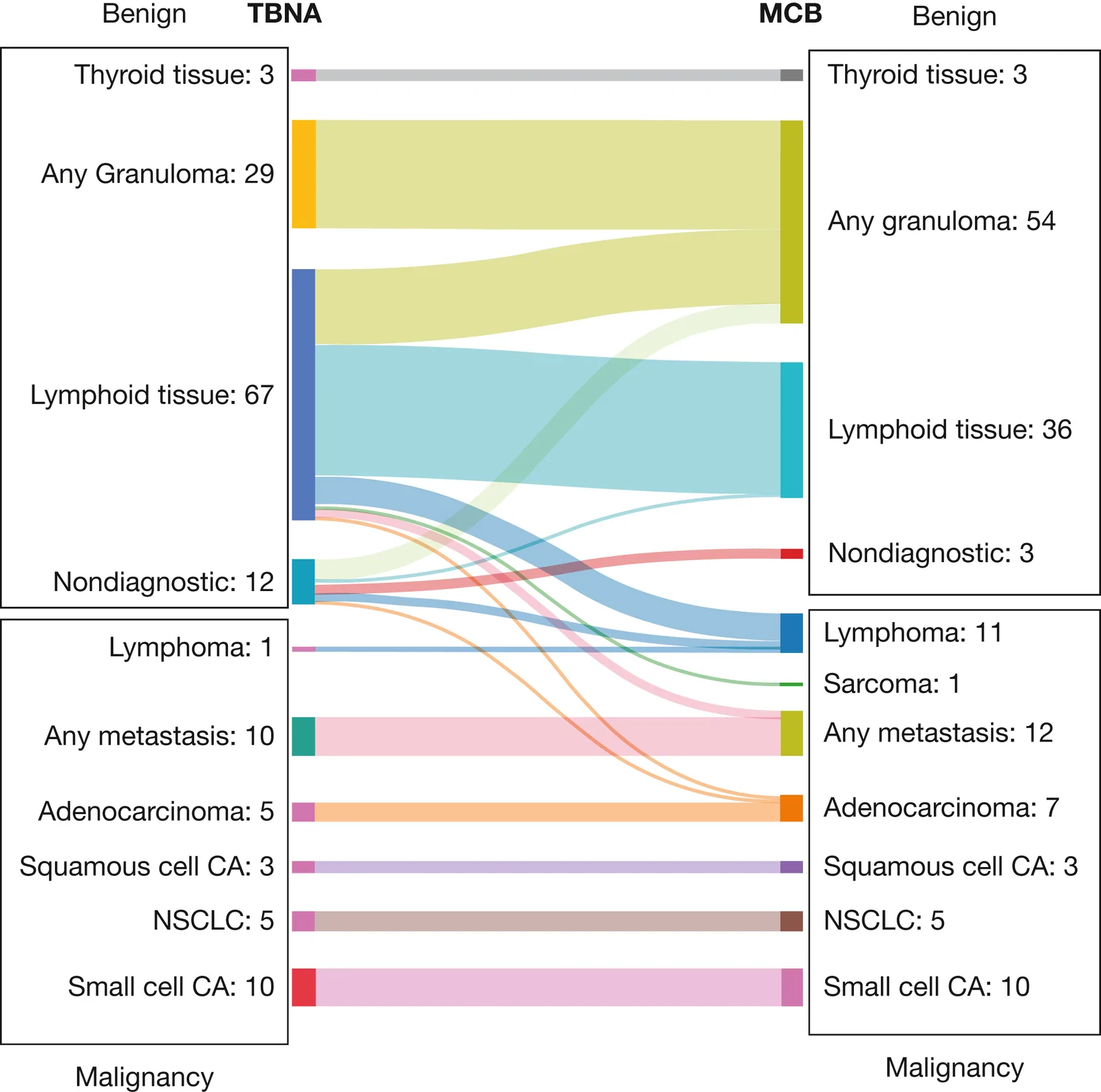
Sankey diagram: changes in diagnostic categories with endobronchial ultrasound-guided TBNA and endobronchial ultrasound-guided MCB. The largest increase in the diagnostic yield of cryobiopsies occurs for any granuloma and lymphoma. MCB = mediastinal cryobiopsy; NSCLC = non-small cell cancer; TBNA = transbronchial needle aspiration; CA = carcinoma. (Sankey diagram flow and color scheme assistance: courtesy of Gary Homeier, APNP-AMCK, and Thomas Gaisl, MD.)

Figure 2 provides an illustration of appropriate immunohistochemical stains performed on a cryobiopsy specimen to diagnose Hodgkin lymphoma. The overall diagnostic performance of EBUS-TBNA and EBUS-MCB is summarized in Table 3 and Figure 3. The diagnostic yield for EBUS-TBNA alone was 91.7% (133 of 145 cases). Among these, EBUS-TBNA successfully diagnosed malignancy in 34 patients. However, EBUS-MCB provided a definitive diagnosis of malignancy in an additional 15 cases, increasing the total number of malignancy diagnoses to 49 cases. Therefore, the diagnostic yield for EBUS-MCB was 97.9% (142 of 145 cases). The diagnostic yield increased significantly from 91.7% (133 of 145) with TBNA alone to 97.9% (142 of 145) with combined TBNA and MCB (McNemar χ^2^ = 9.0, *P* = .003). MCB provided additional diagnostic information in 9 of 12 cases (75%) where TBNA was nondiagnostic, with no cases where TBNA was diagnostic, but the combined approach failed to yield a diagnosis. The false-negative rate for all malignancy was 30.6% (15 of 49 cases). Notably, most false-negative cases were lymphomas, accounting for 91% (10 of 11 cases). For other types of malignancies (excluding lymphoma), the false-negative rate was significantly lower, at 13% (5 of 38 cases).

**Table 3:** Final Histopathologic Diagnosis of EBUS-MCB

Final Histopathology	EBUS-TBNA Alone	EBUS-MCB	Additional Diagnostic Cases With MCB
Benign	**111**	**96**	
Nondiagnostic	12	3	–9^a^
Any lymphoid tissues	67	36	–31^a^
Any granuloma	29	54	25
Thyroid tissue	3	3	0
Malignant	34	49	
Squamous cell CA	3	3	0
Adenocarcinoma	5	7	2
NSCLC	5	5	0
Small cell CA	10	10	0
Metastasis	10	12	2
Lymphoma	1	11	10
High grade sarcoma	0	1	1
Diagnostic yield, % (No.)	91.7 (133)	97.9 (142)	

Data shown for EBUS-TBNA and EBUS-MCB derive from the same 145 patients. Row entries represent the number of cases categorized by each modality within this cohort, rather than distinct patient populations (McNemar χ^2^ = 9.0, *P* = .003). With EBUS-TBNA alone, the diagnosis from the final cytology report showed 111 cases of benign and 34 cases of malignant. However, the final pathology report of EBUS-MCB changed the final diagnosis in 40 cases (25 more cases of nonnecrotizing granuloma and 15 more cases of malignancy). Of these 15 cases, 10 cases had a final pathologic diagnosis of lymphoma. Meanwhile, only 1 case of lymphoma was diagnosed by flow cytometry from an EBUS-TBNA sample. EBUS-MCB = endobronchial ultrasound-guided mediastinal cryobiopsy; EBUS-TBNA = endobronchial ultrasound-guided transbronchial needle aspiration; CA = carcinoma; NSCLC = non-small cell cancer.

a The reduced cases that were redistributed to other, more definitive pathologies.

EBUS-TBNA identified benign conditions in 111 patient. However, among these, 15 patients were ultimately diagnosed with malignancies through EBUS-MCB. Of the remaining 96 cases, EBUS-TBNA detected 29 cases of granulomatous disease, whereas EBUS-MCB identified 25 additional cases, resulting in a total of 54 confirmed granulomatous disease cases. Both techniques were nondiagnostic in 3 patients. One patient had a diagnosis of granulomas by EBUS-TBNA and not by EBUS-MCB. Across the 145 cases where EBUS-MCB was performed, a definitive diagnosis of either granulomatous disease or malignancy was achieved in 106 patients (73.1%) (Table 3). This reflects an additive diagnostic yield for EBUS-MCB over EBUS-TBNA. For the remaining 39 patients with diagnoses of benign lymphoid tissue, or nondiagnostic outcomes, no alternative diagnosis was identified after at least 6 months of clinical follow-up.

We identified 11 lymphoma cases, of which 9 (82%) were de novo. All were successfully diagnosed with EBUS-MCB (100%), whereas EBUS-TBNA yielded a diagnosis in only 1 case (9%). Importantly, none of these patients underwent mediastinoscopy for additional tissue sampling. A breakdown of cases and site-specific flow cytometry submissions is provided in Table 4.

**Table 4:** Summary of Lymphoma Cases: Flow Cytometry and EBUS-MCB Diagnosis

Patient No.	Lymphoma Case Type	Flow Cytometry TBNA	Final Diagnosis EBUS-MCB
VCU 1	De Novo	Not sent	DLBCL
VCU 2	De Novo	Sent: negative	DLBCL
VCU 3	Relapsed	Sent: negative	B-cell lymphoma
VCU 4	De Novo	Sent: negative	B-cell lymphoma
VCU 5	De Novo	Sent: negative	DLBCL
VCU 6	Relapsed	Not sent	Hodgkin lymphoma
VCU 7	De Novo	Sent: negative	DLBCL-PTLD
AMCK	De Novo	Sent: positive	DLBCL
UMSC	De Novo	Sent: negative	DLBCL
WVU1	De Novo	Sent: negative	DLBCL
WVU2	De Novo	Sent: negative	DLBCL

AMCK = Advocate Aurora Medical Center Kenosha; DLBCL = diffuse large B-cell lymphoma; EBUS-MCB = endobronchial ultrasound-guided mediastinal cryobiopsy; PTLD – posttransplant lymphoproliferative disorder; TBNA = transbronchial needle aspiration; UMSC = University Medical Center of Southern Nevada; VCU = Virginia Commonwealth University; WVU = West Virginia University Camden Clark Medical Center.

With 145 paired observations, the study had sufficient precision to detect an absolute difference in diagnostic yield of approximately 6 to 8 percentage points between paired sampling approaches, assuming 2-sided α = .05.

There were no major complications observed during or after the procedures, underscoring the safety profile of EBUS-MCB. Minor bleeding occurred in some cases, effectively controlled with therapeutic suction and cold saline flushes, which are standard measures in routine care. Importantly, there were no instances of serious complications such as pneumomediastinum, pneumothorax, or mediastinitis reported during the brief postoperative period.

## Discussion

The use of EBUS-MCB as a modality for obtaining mediastinal or hilar tissue from lymph nodes has been on the rise. A meta-analysis^11^ comparing EBUS-MCB and EBUS-TBNA showed 10 studies with > 500 patients undergoing EBUS-MCB. The incidence of complications with EBUS-MCB was noted to be the highest, with mild self-limited bleeding at 36%, with only 0.7% (n = 4) needing some bronchoscopic intervention to stop the bleeding. There is also a lot of variation in the methodology used for performing EBUS-MCB. Varied methods, from using a needle track technique for the disposable or nondisposable 1.1- or 1.7-mm ERBE cryoprobe insertion to the use of a high-frequency hybrid knife and neodymium-doped yttrium aluminum garnet lasers to create a track for cryoprobe insertion,^11^^,^^12^ have been described.

Our study was conducted across multiple centers using a single-needle track insertion technique, as previously described, performed by different operators.^13^^,^^14^ This approach represents the simplest and most cost-effective method for performing EBUS-MCB. By visualizing the EBUS-TBNA needle track and puncture site under direct EBUS guidance, the cryoprobe can be precisely inserted with fewer procedural steps to obtain cryobiopsy samples from the target lesion or lymph node. This streamlined technique minimizes complexity while maintaining efficiency. Additionally, it is cost-effective because it involves minimal disposable costs associated with the EBUS-MCB procedure. This method has been widely adopted in various studies worldwide, demonstrating its practicality, affordability, and effectiveness in diverse clinical settings.^9^^,^^15^, ^16^, ^17^, ^18^ An important fact to reiterate is that the randomized controlled trials done with EBUS-MCB^4^^,^^5^ use a high-frequency hybrid knife to insert the cryoprobe. This did not result in a superior diagnostic yield. In our study, EBUS-MCB had a diagnostic yield of 97.9% vs 91.7% for EBUS-TBNA with the simple technique we used for this study.

The number of EBUS-MCB passes and the duration of cryofreeze required to obtain a suitable biopsy specimen are also variable^11^ in multiple studies. A recent study by Kho et al^19^ provides great insight into this variability problem from various studies. It did show a maximum of 3 EBUS-MCB passes to help obtain optimal specimens with a 3- to 4-second cryofreezing time. It also showed a longer freezing time did not help achieve a larger size of the specimen. The size of the optimal specimen obtained in this study^19^ was 4 to 6 mm for a confirmed diagnosis. In our study, the mean aggregated size of the specimen obtained with a mean of 3 EBUS-MCB passes was 9 mm. This reinforces the fact that increasing the number of EBUS-MCB passes may not increase diagnostic yield of the procedure itself.

In our study, EBUS-MCB was performed only in cases where ROSE was inconclusive or when EBUS-TBNA yielded insufficient tissue for a definitive diagnosis, as determined by the cytopathologist. It is prudent to use this approach as initially depicted by Maturu et al.^9^ This approach defines the role of EBUS-MCB as an adjunct tool with EBUS-TBNA and not as a replacement for EBUS-TBNA. The lymph nodes sampled with EBUS-MCB had a mean short-axis diameter of approximately 20 mm. This reflects a more favorable setting because the technique can be more challenging in smaller nodes (< 10 mm) and in regions adjacent to major vascular structures. To minimize risk, proximity to blood vessels was carefully assessed in all cases, and color Doppler was applied when necessary to confirm a safe trajectory before biopsy. Future studies will be important to evaluate the safety and diagnostic yield of EBUS-MCB in smaller lymph nodes, which more closely reflect real-world practice; however, these procedures should be approached with caution and performed by highly experienced operators.

Although EBUS-MCB enhances diagnostic yield in lymphoproliferative diseases, rare thoracic malignancies, and certain benign disorders,^4^^,^^5^^,^^8^^,^^11^^,^^20^ it has shown minimal incremental benefit in non-small cell lung cancer. However, in our study, EBUS-MCB demonstrated a diagnostic yield of 100%, compared with 87% for EBUS-TBNA, for thoracic malignancies excluding lymphomas. For lymphoproliferative disorders, EBUS-MCB had a diagnostic yield of 100% compared with 9% for EBUS-TBNA. This low yield for EBUS-TBNA was because almost all cases in our study (9 of 11) were de novo cases of lymphoma. We also note that our de novo diagnostic yield for lymphoma was < 30% to 60%^21^ reported in the current literature. This is likely due to a smaller sample size of lymphoma cases in the cohort. For granulomatous disorders, the EBUS-MCB technique increased the diagnostic yield by 47% compared with EBUS-TBNA alone.

In our workflow, flow cytometry was not uniformly collected when lymphoid-rich samples were seen on ROSE, with preference often given to obtaining EBUS-MCB specimens for definitive diagnosis. This strategy contributed to the high diagnostic yield observed in our study, and notably, none of the patients required mediastinoscopy for additional tissue sampling. Although flow cytometry can aid in establishing lineage and clonality in de novo lymphoma, particularly in non-Hodgkin subtypes, it does not obviate the need for core tissue sampling with EBUS-MCB, which remains essential for definitive subclassification and for diagnosing entities such as Hodgkin lymphoma. In contrast, flow cytometry has greater utility in relapsed lymphoma, where it can rapidly confirm recurrence without necessitating additional invasive procedures. Looking forward, incorporating a standardized triage approach that includes routine flow cytometry in lymphoid-rich aspirates may further enhance diagnostic efficiency and, in selected cases, reduce the need for supplementary procedures such as cryobiopsies.

A post hoc power calculation was conducted to evaluate whether our sample size was adequate to detect the observed difference in diagnostic yield between TBNA alone and combined TBNA and MCB. Using McNemar test for paired proportions with a 2-sided alpha of .05, our study of 145 patients achieved > 99% statistical power to detect the observed 6.2 percentage point improvement in diagnostic yield (from 91.7% to 97.9%). Smaller differences may not be reliably detected and should be interpreted cautiously.

Our study is unique in its inclusion of multiple centers, encompassing both university hospitals and community hospital settings. It highlights that any high-volume EBUS-TBNA center with a skilled operator can effectively learn and safely perform the EBUS-MCB procedure. Although EBUS-MCB can be conducted by a single operator, as demonstrated in our study, a 2-operator approach^14^^,^^16^ is preferable for optimizing workflow and enhancing safety because the cryobiopsy specimen can be rapidly retrieved and the scope reintroduced in the airway to check for bleeding. In cases where EBUS-TBNA yields are insufficient, EBUS-MCB serves as a valuable adjunct to obtain adequate and representative tissue samples for diagnosis.^15^ Furthermore, to our knowledge, this study represents 1 of the largest series of EBUS-MCB procedures performed across diverse practice settings in the US, demonstrating the feasibility and generalizability of this approach in both academic and community care environments. In terms of complications, our findings are consistent with previous studies, confirming that bleeding is the most common complication associated with EBUS-MCB. However, this bleeding is typically self-limiting and does not require additional intervention, underscoring the safety of the technique.

Although a formal cost analysis was not an objective of this study, a cost review at one participating site showed the following: the disposable cost of an EBUS-TBNA needle was $180 compared with $407 for a cryoprobe, a 126% increase. When both were used in the same procedure ($587), the disposable expense increased by 226% over EBUS-TBNA alone. Although these costs are higher, they remain considerably lower than the operating costs reported for mediastinoscopy,^22^ suggesting a potential economic advantage for EBUS-based approaches.

The limitations of our study include the potential for selection bias due to its retrospective design, which may influence the generalizability of the findings. Additionally, the involvement of multiple levels of experience with operators introduce inherent interoperator variability, which was not specifically accounted for and could potentially affect the diagnostic yield of both EBUS-TBNA and EBUS-MCB. Our sequential, nonrandomized design limits our ability to determine the independent diagnostic performance of EBUS-MCB. Future prospective randomized trials comparing procedure order or sampling strategies are needed to definitively establish the incremental value of each modality. Nonetheless, the involvement of trainees who are interventional pulmonary fellows with varying levels of experience highlights that EBUS-MCB is a technique that, with appropriate training, can be taught, learned, and performed safely. Another limitation is the lack of a detailed comparison between EBUS-TBNA and EBUS-MCB because each patient underwent both components based on inconclusive ROSE findings. This comparative analysis was beyond the primary scope of our study. Future research focusing on standardizing techniques for both experienced and novice operators, and addressing operator variability, may provide further insights into optimizing diagnostic outcomes.

## Interpretation

EBUS-MCB serves as a valuable adjunct to the EBUS-TBNA procedure, potentially improving diagnostic yield, particularly for challenging cases such as lymphoproliferative and granulomatous disorders. Its ability to improve diagnostic yield makes it an important addition to the armamentarium of minimally invasive diagnostic tools. The retrospective nature of our study limits direct comparison between EBUS-MCB and EBUS-TBNA, and we acknowledge this constraint. Further research is needed to evaluate the cost-effectiveness of incorporating EBUS-MCB into routine practice and to standardize the procedural technique. Prospective, head-to-head studies are warranted to clarify the comparative performance of both modalities and better define their respective clinical roles. Additionally, the procedural technique for EBUS-MCB requires further studies to ensure its standardization and optimize its use across diverse practice settings. For centers struggling with low diagnostic yields from EBUS-TBNA, EBUS-MCB offers a safe and effective alternative when performed by experienced, trained operators. Importantly, although the complication rate remains very low, the procedure should not be generalized indiscriminately, because inappropriate use without adequate training and expertise could compromise patient outcomes. When used judiciously and in the hands of skilled operators, EBUS-MCB remains a reliable and feasible option to enhance tissue sampling in complex diagnostic cases.

## Funding/Support

The authors have reported to *CHEST Pulmonary* that no funding was received for this study.

## Financial/Nonfinancial Disclosures

None declared.
